# Naive CD8+ T cells from ART respond to primary vaccination against autologous HIV-1 antigen

**DOI:** 10.1186/1742-4690-9-S1-P16

**Published:** 2012-05-25

**Authors:** Kellie N Smith, Robbie B Mailliard, Weimin Jiang

**Affiliations:** 1 University of Pittsburgh School of Medicine, Department of Molecular Virology and Microbiology, Pittsburgh, USA; 2 University of Pittsburgh Graduate School of Public Health, Department of Infectious Diseases and Microbiology, USA

## Introduction

Antiretroviral therapy (ART) decreases HIV-1 viremia and AIDS-associated mortality. Despite this, HIV infected patients are unable to clear virus during treatment interruption due to insufficient cytotoxic T cell (CTL) activity against the autologous reservoir. It is unclear if naïve T cells from patients on ART can respond to immunotherapies that induce CTL specific for their own, unique virus. Unfortunately, late-evolving virus and the ART reservoir contain escape epitope variants that confer a lack of CTL control. We hypothesize that a dendritic cell (DC)-based immunotherapy during ART can induce CTL capable of eliminating the autologous reservoir, despite their failure to do so during natural infection.

## Materials and methods

We use a naïve T cell flow cytometry panel to evaluate changes in the naive CD4+:CD8+ T cell ratio before seroconversion, during untreated infection, and after ART in an HIV infected subject. We then use this panel to isolate naive CD4+ and CD8+ T cells from this patient during ART and from HIV negative donors. These purified naive T cells are then used in an in vitro model of dendritic cell (DC) vaccination at their in vivo ratios to induce primary IFNγ-producing CTL against autologous HIV-1 Gag, Env, and Nef peptide antigens derived from ART.

## Results

**Figure 1:**
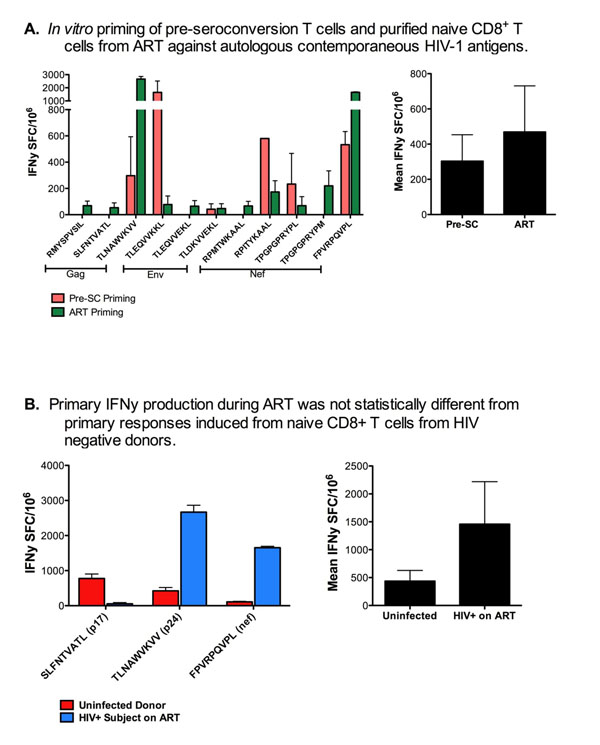


Although partial immune reconstitution occurs during ART, we observed a disproportionate recovery in the naïve CD4+:CD8+ T cell ratio compared to pre-infection. Despite this, we show that naïve CD4+ and CD8+ T cells from ART, when primed at their skewed in vivo ratio against late-acquired, "escape" epitope variants, differentiate into IFNγ-producing CTL comparable to those induced in pre-seroconversion T cells. Additionally, we show that primary CTL responses induced during ART are comparable to those observed in HIV negative donors. Figure 1.

## Conclusion

These data indicate that, despite a disproportionate recovery in the naive CD4+:CD8+ T cell ratio, DC vaccination of naïve T cells from ART can induce CTL specific for autologous "escape" HIV-1 variants, and that these naive T cells can respond to primary vaccination at a level similar to pre-infection. These data support the use of DC immunotherapies in HIV infected patients on ART.

